# Unusually High Incidence of Paediatric Coeliac Disease in Sweden during the Period 1973 – 2013

**DOI:** 10.1371/journal.pone.0144346

**Published:** 2015-12-11

**Authors:** Dimitrios Tapsas, Elisabet Hollén, Lars Stenhammar, Karin Fälth-Magnusson

**Affiliations:** 1 Department of Clinical and Experimental Medicine, Division of Paediatrics, Linköping University, Linköping, Sweden; 2 Department of Clinical and Experimental Medicine, Linköping University, Linköping, Sweden; 3 Division of Microbiology and Molecular Medicine, Linköping University, Linköping, Sweden; 4 Department of Paediatrics and Department of Clinical and Experimental Medicine, Linköping University, Norrköping, Sweden; 5 Department of Paediatrics and Department of Clinical and Experimental Medicine, Linköping University, Linköping, Sweden; Centro di Riferimento Oncologico, IRCCS National Cancer Institute, ITALY

## Abstract

**Objective:**

The prevalence of coeliac disease in Sweden during the “epidemic period” (1984−1996) was one of the highest in the world. The aim of this study was to assess the coeliac disease incidence in our region over the 41-year period, and how diagnostic activity and diagnostic accuracy were affected by the introduction of antibody testing. We also looked into how patients with mild enteropathy were evaluated.

**Methods:**

In the county of Östergötland in Sweden, 2790 paediatric patients were investigated for suspected coeliac disease between 1973 and 2013. Notes were scrutinised for data on sex, age, histopathological reports and final diagnosis. For comparative purposes this period was divided into three sub-periods (1973−1983, 1984−1996 and 1997−2013) named pre-epidemic, epidemic and post-epidemic.

**Results:**

Coeliac disease diagnosis was received by 1,030 patients. The peak incidence rate, 301 cases/100,000 in 1994 for the age group 0−1.9 years is the highest figure ever reported. The other age groups, 2−4.9, 5−14.9, and 15−17.9 years, also had high incidence rates. After the 1984−1996 “epidemic period” the incidence decreased for the youngest group but continued to increase for the other groups. The cumulative incidence at 18 years-of-age for children born during the epidemic reached 14 cases/1000 births, the highest figure hitherto reported. Diagnostic activity differed significantly between the three sub-periods (p<0.001) increasing gradually from 1984 and reaching a peak value of 0.87 in 2012. Cases of mild enteropathy were more frequently regarded as non-coeliac disease cases, decreasing significantly in the “post-epidemic” period (p<0.001).

**Conclusions:**

The incidence rate and cumulative incidence of coeliac disease were possibly the highest ever reported. Changes in diagnostic activity and accuracy could not be attributed to the introduction of new antibody tests, possibly because of other changes e.g. variations in the symptoms at presentation and improved knowledge of the disease among parents and health professionals.

## Introduction

Coeliac disease (CD) is an autoimmune disorder, caused by permanent intolerance to gluten present in wheat, rye and barley, leading to chronic small bowel enteropathy in genetically predisposed individuals [1]. Initially diagnosis of the disease was based solely on the patient’s clinical picture. In 1957, the introduction of the small bowel biopsy technique gave access to mucosal specimens enabling confirmation of the diagnosis [2], and all patients with obvious symptomatology underwent this procedure. Following the introduction of serological testing for antibodies towards gliadin (AGA), endomysium (EMA), and tissue transglutaminase (t-TGA), patients with less overt symptoms also underwent small bowel biopsy and many were diagnosed as having CD [3–5]. Until 2012 the diagnosis of CD in Europe was based on small bowel biopsy according to the criteria laid down by the European Society of Paediatric Gastroenterology, Hepatology and Nutrition (ESPGHAN) [6]. Since then new guidelines have accepted that diagnosis of the disease may be made without a biopsy in certain cases [7].

Over the last four decades, three different grading systems have been employed for histological classification of CD mucosal enteropathy in Sweden; the Alexander, the Marsh, and the Swedish KVAST [8–10]. The evaluation of cases of mild enteropathy, i.e. Alexander Grade II, Marsh Grades 1 and 2, and KVAST borderline, is challenging, and reported disagreement between pathologists in these cases [11].

Between 1984 and 1996, one of the highest observed prevalences of CD worldwide, i.e. 3% among 12-year-olds born in 1993, was reported [12]. This is often referred to as the Swedish “coeliac epidemic” period, a description that we hereafter use in this report without quotation marks. During this period the incidence rate of CD, detected clinically in children under the age of 2 years, was four times higher than in the pre- and post-epidemic periods. The incidence rate for paediatric CD varies worldwide, but studies indicate a clear increase in many countries over recent years [13–16], and there is a clear female dominance [17, 18].

The aim of this study was to see if the incidence of paediatric CD in our region continues to be one of the highest. We also investigated whether diagnostic activity and accuracy of small bowel biopsy increased after the introduction of new screening methods for CD, i.e. serological tests. We also looked at how patients with mild enteropathy, i.e. Alexander Grade II, Marsh Grades 1 and 2, and KVAST borderline were evaluated over the whole period 1973−2013.

## Material and Methods

### Patients

The present report is a cohort study from 1973 to 2013 describing the investigation of suspected CD in a paediatric population in a defined geographical area with stable population and well-developed healthcare system. Patients included in the study resided in the county of Östergötland, in southeast Sweden, served by three paediatric clinics; Linköping, Norrköping and Motala. Throughout the period, the paediatricians caring for CD patients kept a close contact with each other through regular meetings to discuss clinical and scientific issues related to coeliac disease. The county had 437,848 inhabitants, representing almost 5% of the Swedish population, including 87,374 children under the age of 18 years (January 1, 2014). The mean number of births per year during the study period was 4,636 (range 3,920−5,806). All population data were obtained from the Swedish Central Bureau of Statistics. In 1973, a regional CD register was established in the county comprising data on all paediatric patients being investigated under the suspicion of CD. Data on biopsy results, age of the child at biopsy, clinical symptoms etc., were filed at the time of the first biopsy. For the purposes of this study, original notes were re-examined if any data were missing in the files.

Patients who were included in the study fulfilled the following criteria: investigation for suspected CD based on small bowel biopsy or diagnosis based on the revised ESPGHAN criteria; and age below 18 years at the primary biopsy [6].

**Fig 1:**
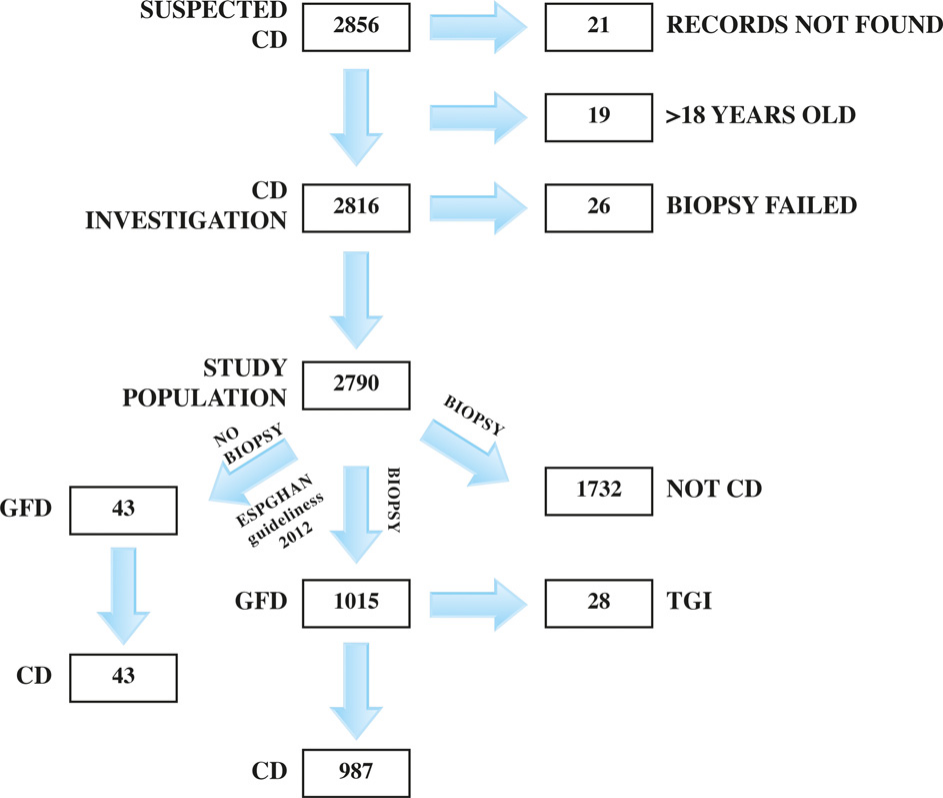
Flowchart of investigation.

Initially, 2,856 patients were included in the study. After further analysis of the register data 66 patients were excluded because they did not fulfil the inclusion criteria for the following reasons: missing information and absence of records to complete the missing data (N = 21); age >18 years (N = 19); and failure to obtain a sufficient biopsy specimen in the period when biopsy was mandatory for diagnosis (N = 26) (Fig 1). For the 41-year study period, 2,790 patients could be included in the final study analysis. The subjects were categorised in three different groups based on the final diagnosis. In the first group, CD was established in 1,030 patients, and among these 987 received a biopsy-confirmed diagnosis while 43 were diagnosed according to the revised ESPGHAN criteria without small bowel biopsy. In the second group, 1,732 were classified as non-CD patients, and in the third 28 were given the diagnosis transient gluten intolerance (TGI), as used in previous classifications (Fig 1). TGI was based on the following criteria: initial biopsy revealing CD, normalisation of small bowel mucosa on a gluten-free diet (GFD) followed by maintained normal biopsy and serological markers after at least two years of gluten challenge [6, 7]. The TGI diagnosis is no longer used [1].

### Histopathological evaluation

Detailed biopsy reports regarding the severity of mucosal alterations were available for 99.9% of the patients who had undergone a small bowel biopsy. During the first years biopsy specimens were taken with a Watson capsule, later with a Storz capsule, and finally at gastroscopy.

The biopsy specimens were initially classified according to the Alexander grading system [9] and subsequently by the Marsh [9] and KVAST [10] systems. Those with Alexander Grade III or above, Marsh Grades 3 or above, and KVAST partial or more serious villous atrophy were considered to be CD [9, 10]. Twenty-nine patients did not fulfill those criteria but were still allocated to the CD group after the paediatrician’s judgment based on a combination of the following factors: 1) convincing clinical symptoms; 2) increased levels of intraepithelial lymphocytes (IELs) in the biopsy specimen; 3) raised antibody levels; and/or 4) little gluten exposure prior to the biopsy.

### Antibodies

The antibody tests, i.e. AGA, EMA, and t-TGA, were performed at the same county laboratory with repeated internal quality assessment. AGA [19] was introduced into clinical practice in the mid 1980:s and EMA in the mid 1990:s [20]. In 1989 and 1999 more than 50% of patients with suspected CD were tested for the antibody in question. In 2003 t-TGA [5] was included in routine CD investigation and applied in more than 50% of cases.

### Ethics

The study was carried out with the approval of the Human Research Ethics Committee of the Faculty of Medicine and Health Sciences, Linköping University, Sweden. All patients were below 18 years of age making the parents their legal representatives, and as such, the parents signed the informed consent. However, a special information sheet was available for the patients/children with ability to read, and those children often co-signed the informed consent to confirm their approval.

### Statistical analyses

Descriptive statistics were reported as means with 95% confidence interval (CI). The chi-square test was used for frequency comparisons of categorical variables. Differences between group means of normally distributed variables were determined by one-way ANOVA and confirmed by LSD post hoc tests. In case of not normally distributed variables, the differences between group means were determined by the Kruskal-Wallis test. A p value less than 0.05 was considered statistically significant. Analyses were performed using SPSS software (SPSS Statistics for Windows, Version 22.0. IBM Corp., Armonk, NY).

The annual incidence rate was defined as the number of new cases diagnosed in an age group over a one-year period, divided by the population of the same age group. The cumulative incidence for a specific birth cohort at a certain age was determined as the number of cases diagnosed in the cohort up to that age divided by the number of births in the cohort. Diagnostic activity was defined as the number of children who underwent a small bowel biopsy per 1,000 children in the county during a specific year. Diagnostic accuracy was specified as the number of CD cases per total number of children with suspected CD who underwent a small bowel biopsy during a specific year.

## Results

### Age and sex distribution

The mean age of the total CD population at the primary biopsy or when the 2012 revised ESPGHAN guidelines were applied, was 6 years (5.7−6.3). The mean ages of the CD children during the pre-epidemic (1973–1983), epidemic (1984–1996) and post-epidemic (1996–2013) periods were 2.2 (1.6−2.8), 2.8 (2.4−3.2), and 8.2 (7.8−8.6) years, respectively (Table 1).

**Table 1:** Mean age of the CD and non-CD patient groups, for three different periods, presented as mean age in years (95% CI).

	Pre-epidemic^1^	Epidemic^2^	Post-epidemic^3^	p value^4^
**CD population**	2.2 (1.6−2.8) n = 98	2.8 (2.4−3.2) n = 319	8.2 (7.8−8.6) n = 613	<0.001
**Non-CD population**	4.2 (3.7−4.7) n = 361	4.5 (4.2−4.8) n = 929	6.3(5.8−6.8) n = 442	<0.001
**p value** ^4^	<0.001	<0.001	<0.001	

^1^ pre-epidemic period = 1973−1983

^2^ epidemic period = 1984−1996

^3^post-epidemic period = 1997−2013

^4^ differences in means were tested with 1-way ANOVA test. The p values in the horizontal axis reveal differences between the three subperiods in both CD and non-CD populations while p values on the vertical axis represent differences between the CD and non-CD populations in each of the three subperiods.

The mean age of patients not being diagnosed with CD was 4.9 years (4.7−5.1), 4.2 (3.7−4.7), 4.5 (4.2−4.8), and 6.3 (5.8−6.8), during the pre-epidemic, epidemic and post-epidemic periods, respectively (Table 1).

Further analyses of the data with post hoc LSD t-tests showed a higher mean age in the post-epidemic period compared to the pre-epidemic (p<0.001) and epidemic (p<0.001) periods for both the CD and non-CD groups.

Comparisons between the CD and non-CD groups for each of the three periods of the study, revealed a higher mean age at diagnosis for the non-CD group during the pre-epidemic and epidemic periods (p<0.001). The opposite was the case in the post-epidemic period where the CD patients were found to be significantly older (p<0.001) (Table 1).

The female-to-male distribution was 1.76:1 in the CD group and 0.93:1 in the non-CD group. No statistically significant results were seen concerning sex distribution between the three different study periods for either subpopulation. Moreover, when comparing the sex ratio of the two groups pair-wise for each of the periods, no significant differences were detected (Table 2).

**Table 2:** Female:male ratio of CD and non-CD patients before, during and after the Swedish coeliac epidemic period.

	Pre-epidemic^1^	Epidemic^2^	Post-epidemic^3^	p value^4^
**CD population**	1.5:1 (n = 98)	1.8:1 (n = 319)	1.8:1 (n = 613)	0.17
**Non-CD population**	1:1 (n = 361)	0.89:1 (n = 929)	0.93:1 (n = 442)	0.73
**p value** ^4^	0.48	0.67	0.72	

^1^ pre-epidemic period = 1973−1983

^2^ epidemic period = 1984−1996

^3^post-epidemic period = 1997−2013

^4^ differences in ratios were tested with chi-square test

### Incidence rate

**Fig 2:**
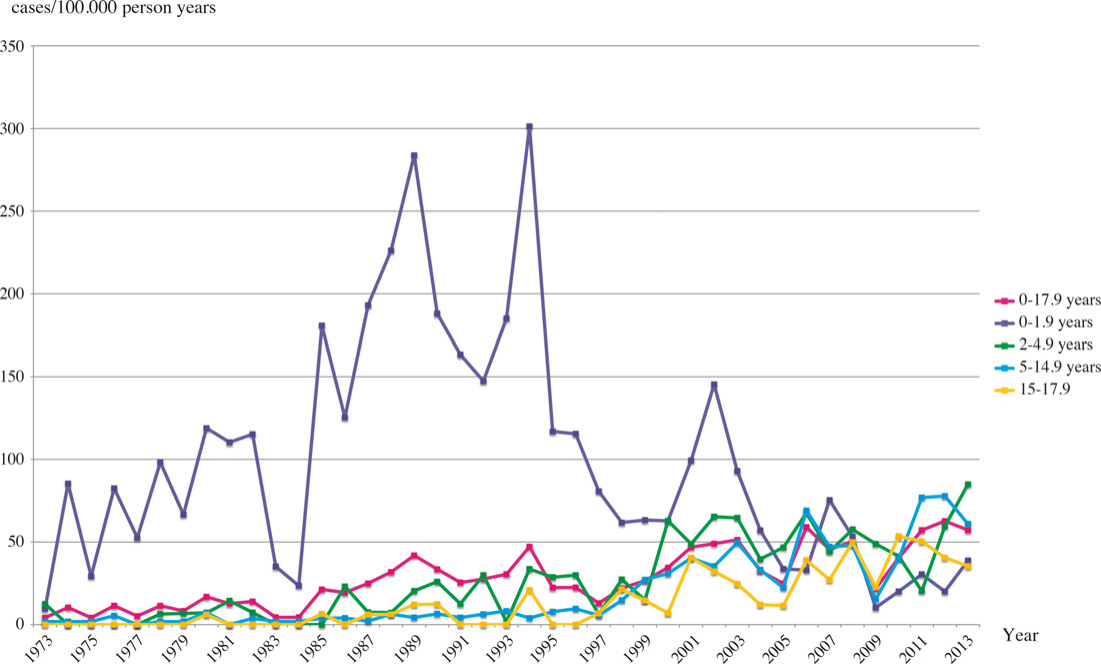
Annual incidence rate of CD in different age groups from 1973 to 2013.

The incidence rate for the age group 0−1.9 years increased drastically during the CD epidemic period in Sweden, reaching a peak value of 301 new cases per 100,000 person years in 1994. After the epidemic period the rate decreased gradually reaching its lowest value in 2009 with only 10 new cases per 100,000 person years (Fig 2).

Older children between 2 and 4.9 years-of-age, showed a gradual increase in incidence rate from the start of the epidemic period, continuing even after it ended. The highest rate was recorded in 2013 with 85 new cases per 100,000 person years, though never reaching the high rates of the youngest group during the epidemic period (Fig 2).

The patient group aged 5−14.9 years was characterised by a steady increase in incidence rate, though noticed later than in the previous group. The peak value for this group was reported in 2012 with 78 new cases per 100,000 person years (Fig 2).

The incidence rate for the adolescence group (15–17.9 years) started to increase in the epidemic period, but there were some years with a total absence of new cases. The maximum number of new cases per 100,000 person years was 54 in 2010 (Fig 2).

The incidence rate for the total CD population increased gradually during the first years of the epidemic period but decreased temporarily at the end of the period and increased again after 1997, reaching its highest value in 2012 with 63 new cases per 100,000 person years (Fig 2).

### Cumulative incidence

**Fig 3:**
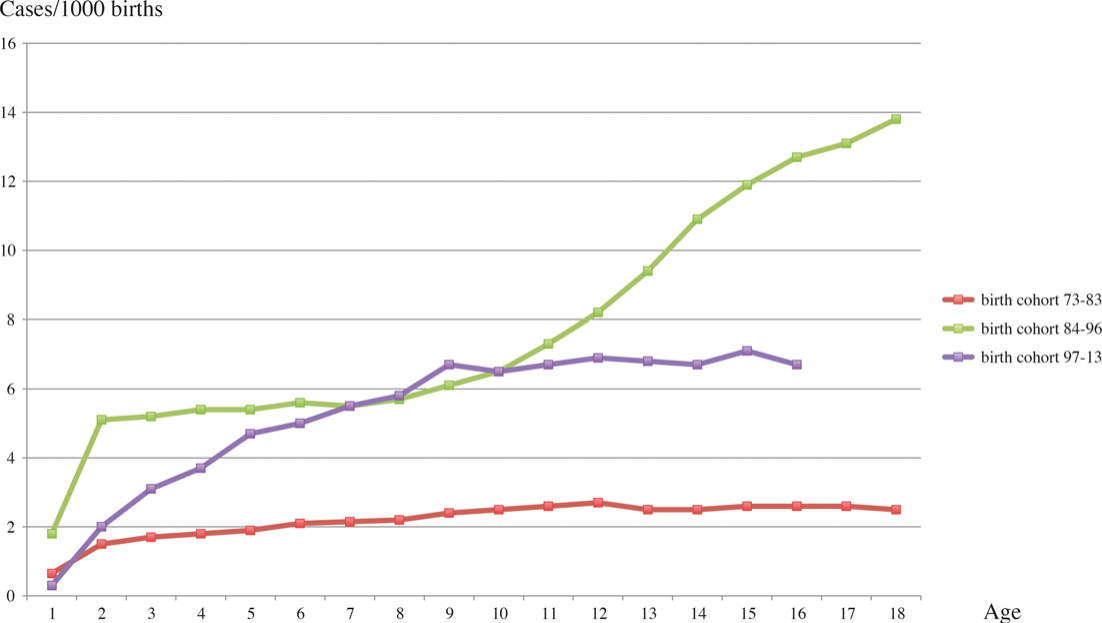
Cumulative incidence of CD by age for the birth cohorts 1973−1983, 1984−1996, and 1997−2013.

The cumulative incidence of CD children born before the epidemic period revealed a clear tendency to increase until 12 years-of-age. After this age the incidence remained stable. The highest result reported for this cohort was 2.7 new cases/1000 births at 12 years-of-age (Fig 3).

The cohort born 1984−1996, i.e. children born during the Swedish CD epidemic period, showed a sharp increase up to 2 years-of-age, then a fairly stable level until 8 years, with another sharp increase in cases, with no plateau, up to 18 years. The cumulative incidence reached the highest value at 18 years-of-age with 13.8 new cases/1000 births (Fig 3).

The youngest cohort displayed a moderate increase up to 9 years but after that the incidence stabilised. The highest cumulative incidence for this particular group was observed at 15 years-of-age with 7.1 new cases/1000 births (Fig 3).

### Diagnostic activity

**Fig 4:**
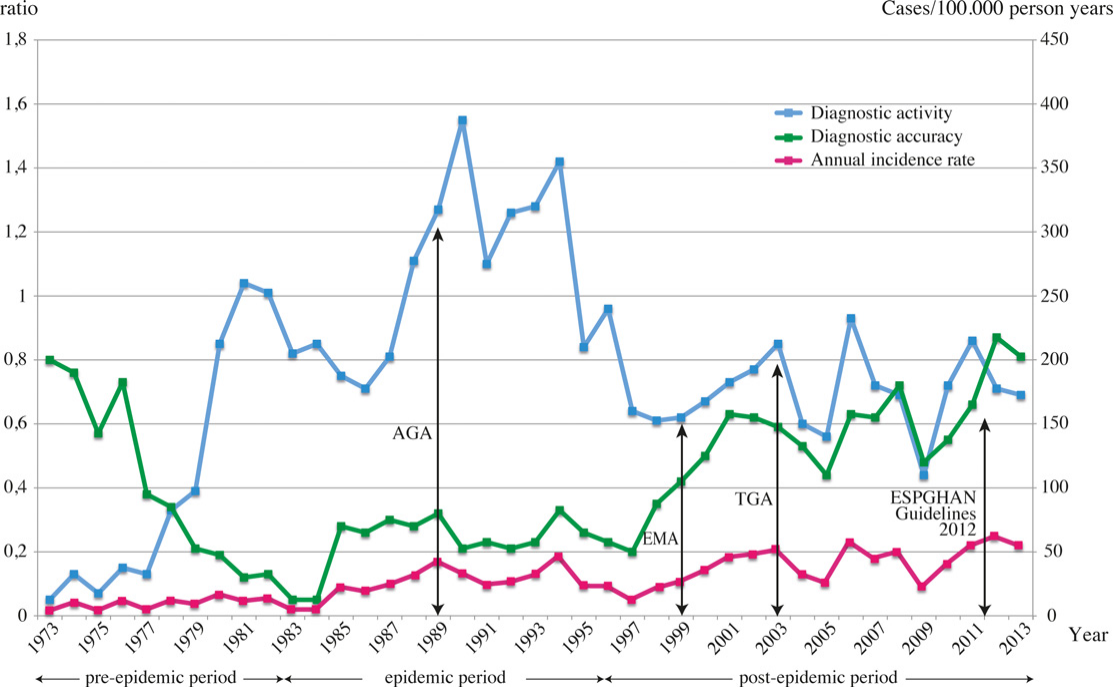
Diagnostic activity and accuracy among Swedish children investigated for CD with biopsy between 1973 and 2013. Arrows indicate when AGA, EMA, t-TGA tests were used in >50% of the cases investigated for CD, and also the introduction of the 2012 ESPGHAN guidelines for CD diagnosis into clinical practice. Diagnostic activity was considered as the number of subjects biopsied per 1,000 children in the study population. Diagnostic accuracy was specified as the number of CD cases per total number of children with suspected CD who underwent biopsy. The diagnostic activity and accuracy are described as ratios. The purple line represents the incidence rate of CD in the pediatric population during the same time period.

During the pre-epidemic period the diagnostic activity, i.e. number of investigated per 1000 children, had a mean value of 0.45, with an increase to 1.07 in the epidemic period falling to 0.69 in the post-epidemic period. The difference between the three periods was significant (p<0.001) (Fig 4).

The diagnostic activity had already increased from the mid 1980:s reaching a peak value of 1.55 in 1990. From 1994 and onwards, both diagnostic activity and incidence rate displayed parallel changes (Fig 4).

### Diagnostic accuracy

The mean values for diagnostic accuracy, i.e. the proportion of children receiving a CD diagnosis among those undergoing primary biopsy, were 0.38, 0.24 and 0.56, respectively for

the pre-epidemic, epidemic, and post-epidemic periods (Fig 4). The difference between the three study periods was significant (p<0.001). The diagnostic accuracy increased gradually from the beginning of the epidemic period in 1984 reaching a peak value of 0.87 in 2012 (Fig 4). The diagnostic accuracy in 1973 was 0.8, which is almost identical to that in 2013.

### Histopathological evaluations

In the CD group, 61.7% of the histopathological evaluations were assessed using the Alexander grading system, 14.2% the Marsh, 23.9% the KVAST grading system, and 0.2% unknown (Table 3). In 2.9% (29/987) of the patients, CD was diagnosed even though the biopsy revealed mild enteropathy, i.e. Alexander Grade II, Marsh Grades 1 and 2, and/or KVAST borderline. Of these cases, the Alexander grading system was used in 10 cases (1.4% of all Alexander evaluations), the Marsh system in 12 cases (7.4% of all Marsh evaluations), and the KVAST in 10 cases (3.7% of all KVAST evaluations) (Table 4). Three patients had double evaluation (both the Marsh and KVAST manuals) and in one of those cases detailed evaluation was missing. When comparing the three scoring systems the result was statistically significant (<0.001) (Table 4). Pair-wise comparisons concerning cases with mild enteropathy showed significant differences between the Alexander and Marsh systems (p<0.001), and Alexander vs KVAST (p = 0.049). In the CD group the frequency of patients with mild enteropathy was significantly different between the pre-epidemic (n = 2, 2%), epidemic (n = 1, 0.03%), and post-epidemic periods (n = 26, 4.5%) (p = 0.002) (Table 5).

**Table 3:** Frequencies of histopathological evaluations with three different manuals both in the CD and non-CD populations.

	CD, n^1^ (%)	Non-CD, n^1^ (%)
**Alexander evaluations**	695 (61.7%)	1619 (90.7%)
**Marsh evaluations**	161 (14.2%)	55 (3.1%)
**KVAST evaluations**	270 (23.9%)	110 (6.2%)

^1^The total number of histopathological evaluations for both groups is larger in comparison to the total number of CD (n = 987) and non-CD patients (n = 1732) because of double evaluations in some patients with two different manuals

**Table 4:** Frequencies of patients (of the total number of histopathological evaluations for each method) with a mild enteropathy, i.e. Alexander Grade II, Marsh Grades 1 and 2, and KVAST borderline, that were judged as CD or non-CD.

	CD^2^, n (%)	Non-CD^3^, n (%)
**Alexander Grade II**	10 (1.4%)	113 (6.9%)
**Marsh Grade 1, 2**	12 (7.4%)	15 (27.2%)
**KVAST borderline**	10 (3.7%)	19 (17.2%)
**p value** ^1^	<0.001	<0.001

^1^ differences in frequencies were tested with chi-square test

^2^ three patients had double evaluation with the Marsh and KVAST systems

^3^ five patients were evaluated with both Marsh and KVAST systems

**Table 5:** Mild enteropathy (Alexander Grade II, Marsh Grades 1 and 2, and KVAST borderline) in CD, non-CD patients, and in the whole study population, during the three study periods.

	Pre-epidemic^1^	Epidemic^2^	Post-epidemic^3^	p value^4^
**CD children with mild enteropathy** ^5^	2% (n = 2/98)	0.03% (n = 1/319)	4.5% (n = 26/570)	0.002
**Non-CD children with mild enteropathy** ^5^	31.3% (n = 113/361)	1.5% (n = 14/929)	3.6% (n = 16/442)	<0.001
**All children with mild enteropathy** ^5^	25% (n = 115/459)	1.2% (n = 15/1248)	4.2% (n = 42/1012)	<0.001
**p value** ^4^	<0.001	0.169	0.774	

^1^ pre-epidemic period = 1973−1983

^2^ epidemic period = 1984−1996

^3^post-epidemic period = 1997−2013

^4^ differences in frequencies were tested with the chi-square test

^5^ mild enteropathy = Alexander II, Marsh 1 and 2, and KVAST borderline

In the assessment of biopsies classified as non-CD, the Alexander system was used in 90.7% of all the histopathological evaluations, the Marsh in 3.1%, and the KVAST in 6.2%. The evaluation of mild enteropathy, i.e. Alexander II, Marsh 1and 2, and KVAST borderline, was present in 8.2% (142/1732) of the biopsies. Thus the vast majority, i.e. 113 cases (6.9% of all Alexander evaluations), was detected using the Alexander grading system. Additionally, 15 cases were identified with the Marsh system (27.2% of all Marsh evaluations), and 19 cases using the KVAST system (17.2% of the KVAST evaluations) (Table 4). In five cases double evaluation using both Marsh and KVAST systems were made (in one of these cases detailed information was not found). As in the CD group pair-wise comparisons concerning cases with mild enteropathy revealed significant differences between the Alexander and Marsh systems (p<0.001), and between Alexander and KVAST (p<0.001) (Table 4). The frequency of patients with mild enteropathy also differed significantly between the pre-epidemic (n = 113, 31.3%), epidemic (n = 14, 1.5%), and post-epidemic periods (n = 16, 3.6%) (p<0.001) (Table 5).

When comparing number of patients with mild enteropathy regardless of system used for classification during the three study periods, significant differences were found (Table 5). Significantly fewer cases with mild enteropathy were detected in the pre-epidemic period compared to both the epidemic (p<0.001) and post-epidemic periods (p<0.001). The frequency of cases with mild enteropathy was higher in the group of non-CD patients (8.2%) compared to the CD group (2.9%) (p<0.001) in the whole study population.

Among the 142 cases of mild enteropathy at the initial biopsy considered to be non-CD patients, 41 underwent a second small bowel biopsy because of inconclusive findings and remaining high suspicion of CD. After a second biopsy, all these subjects were diagnosed as non-CD patients; 23/41 having a less pathologic histological picture while 18/41 had the same picture. None of the 101 remaining cases of mild enteropathy underwent a repeat biopsy before they were 18 years-of-age.

## Discussion

The present study was conducted in a well-defined geographic area in Sweden and included 2,790 patients who underwent complete CD investigation. The 41-year study period represents the longest series of observations regarding changes in incidence rate and cumulative incidence among Swedish paediatric CD patients, and possibly worldwide. Furthermore, a unique aspect of the study is the fact that patients with a non-CD diagnosis could also be accounted for, which gave us the opportunity to draw conclusions regarding diagnostic activity and accuracy. Most other studies describe CD cases only. During the 41 years covered by this study several CD milestones were passed, including substantial changes regarding diet practice, diagnostic aids including antibody and genetic testing, and substantial improvement in knowledge of the disease among both health professionals and parents.

The mean age of CD patients at diagnosis increased after the epidemic period, a result concurring with other studies reporting mean ages from 6.7 to 9.3 years [18, 21, 22]. In the present study a peak value at 9.9 years was observed (data not shown), one of the highest reported internationally. On the contrary, other studies have reported that the age at diagnosis has not shifted upwards, instead remaining at the same level as in the pre-epidemic and epidemic periods in Sweden [23, 24]. Both genetic background and different diet practice among those populations could contribute to this discrepancy. In the post-epidemic period a shift in age at diagnosis was found between the CD and the non-CD group. Until then, the mean age at diagnosis was lower among the CD patients but in the post-epidemic period the non-CD subjects were significantly younger at diagnosis. This trend has previously been reported in another Swedish study, albeit not a significant difference [25]. The male-to-female distribution among CD subjects showed a female dominance which is well-known and has been reported previously [26, 27].

We also found a remarkable variation in incidence rate among the youngest group (0−1.9 years) with an extreme increase during the epidemic period followed by a decrease the years after, confirming Swedish data from Namatovu *et al*. who studied CD in a larger catchment area with 15–40% national coverage from 1973 to 1997, and 100% the following years [14]. Their study also reported the highest incidence rate in the youngest group with approximately 250 cases/ 100,000 persons in 1994, compared to 301 cases/100,000 persons at the same point in time in the present study. The incidence rate among the older age groups, i.e. 2−4.9, 5−14.9, and 15−17.9 years, began to increase during the epidemic period and continued into the post-epidemic period [14]. The really high incidence levels among the youngest Swedish patients has decreased gradually and this has been attributed to changes in diet recommendations in Sweden such as the time of gluten introduction and the degree of gluten intake [12].

The incidence rate for the whole CD cohort increased during the epidemic period until 1994 and then decreased until 1997 after which a gradual increase was observed reaching a peak value in 2012 with 63 new cases per 100,000 person years. This is the highest reported incidence rate among a Swedish child population (0−17.9 years) in the last four decades. Namatovu *et al*. reported a maximum incidence rate of approximately 50 cases per 100,000 persons until 2009 [14]. An increase of the paediatric CD incidence rate was also observed in other countries/regions such as the U.S.A, Scotland, Wales etc., but has never reached the high levels reported in Sweden [13, 15, 28].

Increased awareness of the disease among professionals and parents [29] and the introduction of serological tests, i.e. AGA, EMA and t-TGA, could have contributed to the high incidence rate among the older CD patients, but this does not explain the steady decrease in the post-epidemic period seen in the youngest group (0−1.9 years).

The cumulative incidence for the pre-epidemic and post-epidemic cohorts increased until 10–12 years-of-age and then remained stable. The post-epidemic cohort, however, had higher cumulative incidence levels throughout the study in comparison to the pre-epidemic cohort. Our results, especially the pre-epidemic cohort, are consistent with those of Namatovu *et al*. [14] and Olsson *et al*., previously reported from Sweden [30]. For the cohort born 1984−1996, i.e. the “epidemic cohort”, we report the highest cumulative incidence figures yet seen in a Swedish paediatric CD cohort with 13.8 cases/1000 births at 18 years-of-age. Prior to that, the highest cumulative incidence in Sweden was approximately 7 cases/1000 births up to 14 years-of-age reported by Namatovu *et al*. [14]. As age increased, the gap in cumulative incidence between the epidemic cohort and the pre-epidemic and post-epidemic cohorts became wider. The cumulative incidence in a Spanish cohort of patients with CD and mean age at diagnosis of 2.3 years, recorded in their national register between 2006−2007 was almost 7 cases/1000 births [24]. Our results, 13.8 cases/1000 births at 18 years-of-age, are presumably the highest cumulative incidence rates ever reported [24, 31, 32]. Even so, it is well-known that CD patients detected by their clinical picture represent approximately 1/3 of all CD cases in the population [12].

Factors contributing to the high levels seen in our region were presumably the active interest shown by one of the authors, LS, who worked with the disease throughout the 41-year period, and the close cooperation between the paediatricians responsible for the care of these patients. The fact that the population was stable during the study period enabled almost complete coverage of the files. Documentation of both diagnostic activity and accuracy, which mirrors active search for the disease, is one example of cooperation in our region. To our knowledge, other groups have not reported these data making comparisons impossible.

Diagnostic activity, i.e. the aptitude to perform small bowel biopsy, increased during the 1970:s presumably due to the fact that small bowel biopsy became an established diagnostic tool for the CD at that time. In the early years of biopsy, only children with pronounced and typical symptoms were investigated, hence the high diagnostic accuracy. At the same time the incidence rate of the disease remained stable and low. In the mid 1980:s an increase in diagnostic activity occurred. This coincided both with introduction of AGA testing, known for high sensitivity, but not so high specificity [33], and large numbers of infants presenting with typical clinical symptoms during the epidemic period. Both these two changes affected diagnostic activity. Some years later, at the end of the epidemic period, diagnostic activity decreased and accuracy increased. By the end of the 1990:s, when the majority of the CD subjects were investigated using EMA testing, which proved to be very specific [33], diagnostic activity increased once more and is now in line with diagnostic accuracy. The same tendencies were observed regarding incidence rate and diagnostic activity after the introduction of TGA testing as a diagnostic tool.

Since 1984 diagnostic accuracy has increased steadily reaching the top value of 0.87 in 2012. Curiously, this is very close to the value observed in 1973, but diagnostic activity was 13.8 times higher and the cumulative incidence 10.3 times higher in 2013 than in 1973.

The clinical evaluation of a histopathological report indicating mild enteropathy, i.e. Alexander Grade II, Marsh Grades 1 and 2, and KVAST borderline, has always been a challenge for the paediatric gastroenterologist. The cases of mild enteropathy represented 6.3% (172/2719) of the whole study population, which is three times higher than in an adult group of 66 patients reported by Mahadeva *et al*. [34]. A retrospective study from the Mayo Clinic reported mucosal changes with increased IELs only in 1.3% of the biopsies, and after a re-evaluation of those cases 10% were classified as CD patients [35]. In the present study, a few patients with mild enteropathy (29/2719, i.e. 1% of the total population) were regarded as CD patients based on their clinical status and serological results. The frequency of biopsies with mild enteropathy in the whole study group decreased significantly after the pre-epidemic period, in contrast to a significant increase in the post-epidemic period. More biopsies were classified as mild enteropathy when the Marsh and KVAST manuals were used than when using the Alexander system. It has previously been shown that agreement between different pathologists concerning Marsh Grades 1 and 2 was poor [11], a fact that could be considered a bias in the evaluation of this patient group in our study. On the other hand, there was greater agreement on Marsh Grades at both ends of the spectrum (Marsh 0 and Marsh 3c) [11].

A Swedish-Danish study of inter-observer variation in assessing paediatric CD biopsy specimens revealed moderate agreement among pathologists [36]. Mubarak *et al*. reported discrepancy between two independent evaluations with under-diagnosis of paediatric CD in 7.4% of cases [37]. Re-evaluation of the complete material in this study by one experienced pathologist would have been the ideal control, but unfortunately this was not feasible, so we have to rely on the internal controls performed regularly at all laboratories in Sweden.

## Conclusions

The incidence rate of CD among Swedish children has continued to increase even after the end of the epidemic period in 1996. The cumulative incidence at 18 years-of -age for the cohort born during the epidemic period reached, to our knowledge, the highest figure ever reported. Both diagnostic activity and accuracy in performing small bowel biopsy in the investigation of CD has increased significantly over the years, but could not clearly be attributed to the introduction of serological tests in clinical practice because of other parallel changes in feeding regimen and diagnostic activities. The cases of mild enteropathy i.e. Alexander Grade II, Marsh Grades 1 and 2, and KVAST borderline, were usually diagnosed as non-CD patients and these decreased over the period of the study.

## Abbreviations

CD: coeliac disease
AGA: anti-gliadin antibodies
EMA: anti-endomysium antibodies
t-TGA: anti-tissue transglutaminase antibodies
ESPGHAN: European Society for Paediatric Gastroenterology, Hepatology and Nutrition
CI: confidence interval
TGI: transient gluten intolerance
GFD: gluten-free diet
IELs: intra-epithelial lymphocytes
